# Private Patients in Grady Hospital

**Published:** 1894-10

**Authors:** 


### PRIVATE PATIENTS IN GRADY HOSPITAL.

There are some paragraphs in the published rules and regulations of Grady Hospital, which have an interest for the professional men of Atlanta, who are not connected with the official staff of that Institution. Under the head of attending Physicians and Surgeons, it is set forth in clause 1, as follows: "No physician, other than those on the Medical Board, shall attend patients in the wards of the hospital. Patients upon being admitted to the private rooms of the hospital, shall have the right to call in the physician or surgeon of their choice, but shall pay the same amount per week for board, room and nursing, as patients under treatment of the medical staff, occupying the same rooms; and to such patients, the hospital shall not be allowed to furnish instruments, surgical appliances, anesthetics, or medicine."

Clause No. 7: "No physician or surgeon shall have in the hospital, at the same time, more than two cases from which he receives a fee. For the admission of any patient, beyond this number, a special permission must first be obtained from the Executive Committee." It is understood that the Medical Board has construed clause No. 1 in such form that any physician or surgeon outside of the regular staff cannot occupy the operating room of the hospital for any surgical work upon a private patient, although the said patient may be paying the highest rate for hospital accommodation.

It is known, also, that the proposition has been made, and is now under consideration by a committee of the Medical Board, to deprive all such private patients of the care of the House Physicians and Surgeons, either as assistants in the performance of surgical operations or in supervising the patients, whether medical or surgical, in the absence of the regular attendant in charge of the case. Thus it turns out that private patients of professional men not belonging to the staff, are placed at a hospital without receiving the benefits which such an institution is designed to confer, and could be as well treated at a private boarding-house with less expenditure.

Under the head of "Private Patients," we learn that the charitable work of the hospital is done in the wards. "A limited number of private rooms have been set apart for those seeking necessary hospital accommodations. The rooms are intended to replace the comforts and conveniences of home. The income received from the board and nursing of private patients is applied toward the support of the constantly increasing charity work of the hospital."

For the purpose of aiding in this laudable undertaking, there are doubtless many practitioners, like the writer, who would cheerfully recommend their patients to avail themselves of the Grady Hospital, if they are allowed the same care and attention under their charge, which are extended to the private patients treated by members of the hospitsl staff in paid quarters. Of course the internal management of the hospital should not be interfered with by outside physicians and surgeons, but whatever facilities for the proper treatment of patients who pay for hospital accommodations that are afforded to members of the staff ought to be extended to the members of the medical profession from without who take their own patients there for treatment.

If the Medical Board would avoid the impression that the private patients of others are placed at a disadvantage in entering the Grady Hospital, they must be granted the same privileges and benefits received by those in charge of the members of the staff. In making a provision that "patients upon being admitted to the private rooms of the hospital, should have the right to call in the physician or surgeon of their choice, but shall pay the same amount per week for board, room, and nursing as patients under treatment of the medical staff occupying the same rooms," the only condition attached is, that "to such patients the hospital shall not be allowed to furnish instruments, surgical appliances, anesthetics, or medicines."

It surely could not have been expected that such patients should be debarred from the benefits of the operating room or the care of the House Physicians and Surgeons. J. M'F. G.

